# Mucopolysaccharidosis Type II Screening, Diagnosis, and Management: A Literature Review and Practical Recommendations for Newborn Screening Programs and Health Care Providers to Support Families and Improve Outcomes

**DOI:** 10.3390/ijns12030066

**Published:** 2026-08-12

**Authors:** Amy Gaviglio, Natasha Bonhomme, Barbara Burton, Norman Matthew Ellinwood, Joseph Muenzer, Kim Stephens, Ravi Pathak, Carolyn Schaeffer-Koziol

**Affiliations:** 1 Connetics Consulting LLC, Minneapolis, MN 55417, USA; 2 Expecting Health, Washington, DC 20016, USA; 3 Edwards Family Division of Genetics and Rare Diseases at Ann & Robert H. Lurie Children’s Hospital of Chicago, Chicago, IL 60611, USA; 4 Department of Pediatrics, Feinberg School of Medicine, Northwestern University, Chicago, IL 60611, USA; 5 National MPS Society, Durham, NC 27709-4686, USA; 6 University of North Carolina at Chapel Hill, Chapel Hill, NC 27516, USA; 7 Takeda Pharmaceuticals USA, Inc., Lexington, MA 02421, USA

**Keywords:** Hunter syndrome, MPS II, mucopolysaccharidosis type II, NBS, newborn screening program, rare diseases

## Abstract

Mucopolysaccharidosis type II (MPS II; also known as Hunter syndrome), is a rare X-linked lysosomal disease that leads to progressive tissue and organ damage. Early treatment is essential as most symptoms of MPS II are not reversible. Consequently, MPS II has been added to many newborn screening (NBS) programs. This narrative literature review provides practical recommendations from a multidisciplinary expert panel on the US-based NBS for MPS II and its diagnosis and clinical management. Recommendations for NBS programs include aiming for universal access to NBS within their jurisdiction, implementing tiered testing to support diagnostic accuracy, and providing infrastructure for confirmatory testing and post-screening support for families. Recommendations for health care providers (HCPs) include communicating test results empathetically and alongside verbal and written information, allowing families to express their feelings, and consulting an MPS II specialist to support treatment recommendations. The NBS programs and HCPs should work together to ensure positive screening results are communicated effectively and to provide equitable access to treatment and long-term care. Such a coordinated and appropriately resourced effort involving NBS programs, HCPs, and patient advocates will ensure better support for families and the best possible outcomes for individuals with MPS II.

## 1. Introduction

### 1.1. Mucopolysaccharidosis Type II

**Figure 1:**
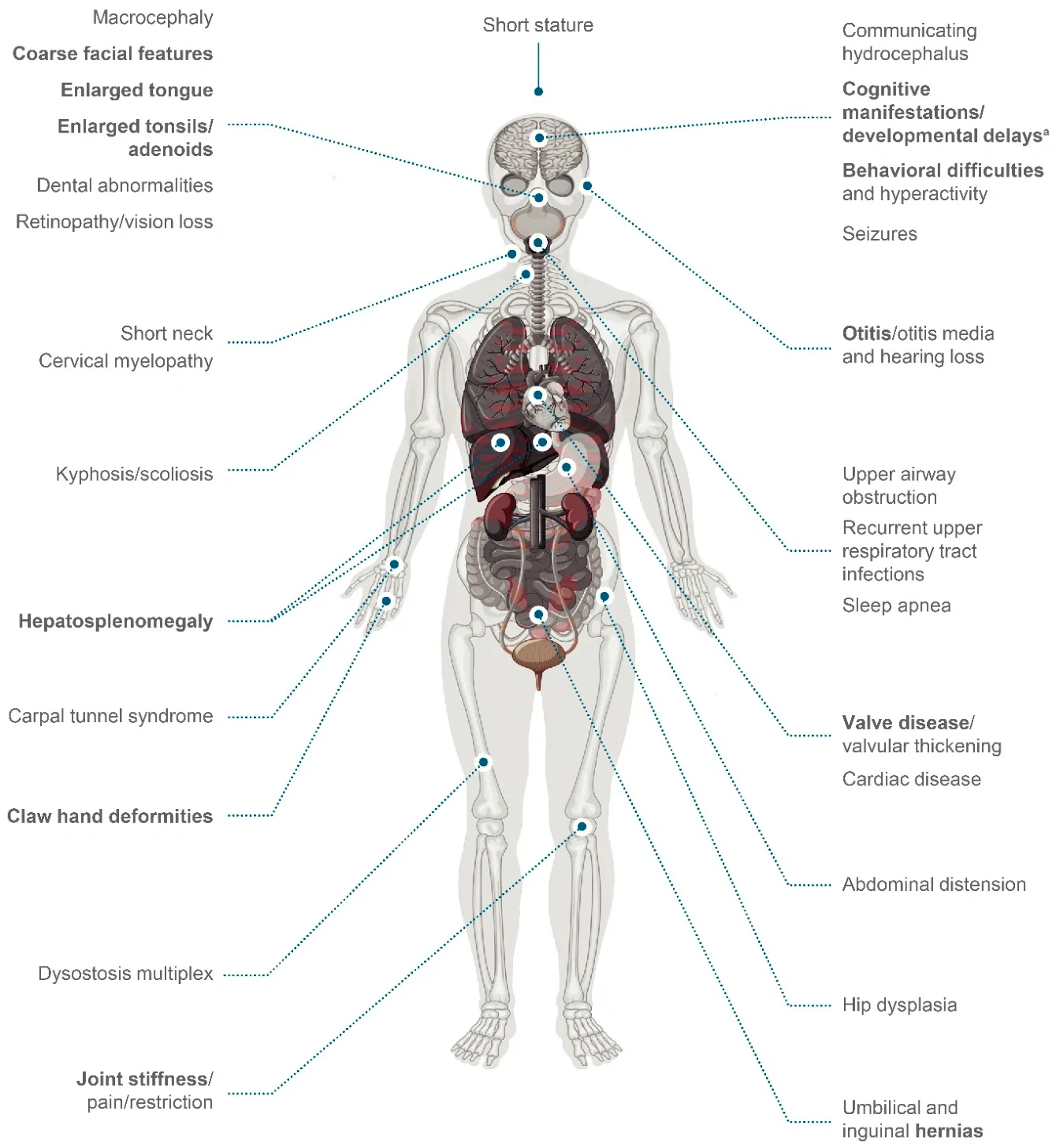
Signs and symptoms of MPS II [3,5,6,9,10]. Signs and symptoms shown in bold were reported in >50% of individuals with MPS II in Wraith et al. [5] and/or Lau et al. [6]. ^a^ Severe cognitive decline/developmental delays characterize the neuropathic presentation of MPS II. Abbreviation: MPS II, mucopolysaccharidosis type II.

Mucopolysaccharidosis type II (MPS II; also known as Hunter syndrome) is a rare X-linked recessive lysosomal disease. The estimated incidence is 1.36 per 100,000 live births in the United States (US), based on two US newborn screening (NBS) studies [1,2]. It is very rarely seen in females [3]. The clinical manifestations of MPS II result from deficient activity of iduronate-2-sulfatase (IDS), which leads to the accumulation of the glycosaminoglycan (GAG) substrates heparan sulfate (HS) and dermatan sulfate (DS), leading to progressive damage to tissues and organs throughout the body [3,4]. There are multiple symptoms and clinical features throughout the body, the most common of which are coarse facial features, enlarged liver and/or spleen, and joint stiffness and/or limitations [5,6] (Figure 1). Children may also present with recurrent respiratory tract or ear infections, behavioral and cognitive changes, and developmental delays [5,6]. Although signs and symptoms occur along a spectrum, MPS type II is usually further typed by the presence or absence of neuropathology, especially cognitive impairment. This distinction within the spectrum of MPS II is termed neuropathic (severe) and non-neuropathic (attenuated) disease, and the presence or absence of neuropathology may inform approaches to care [3]. Both the severe and attenuated presentations of MPS II have somatic involvement, whereas individuals with neuropathic MPS II develop severe cognitive impairment [3]. Pathological signs of abnormal GAG storage in the central nervous system (CNS) can be seen as early as 23 weeks of gestation [7]; however, signs and symptoms of MPS II generally appear between 18 months and 4 years of age [8]. Symptoms typically appear earlier in the neuropathic presentation of MPS II compared with the non-neuropathic presentation (averaging 1.5 and 2.2 years, respectively, based on an analysis of the Hunter Outcome Survey [6]). Regardless of the age of symptom presentation, individuals and their families usually experience a long diagnostic odyssey between clinical onset and diagnosis. Disease manifestations vary across individuals and disease severity [3,5,9,10]; however, clinical manifestations are generally similar across affected family members [11,12,13]. Based on the Hunter Outcome Survey global registry, without treatment, age at death ranges from approximately 6 to 23 years for individuals with neuropathic MPS II and 5 to 45 years for individuals with non-neuropathic MPS II [14]. However, individuals with non-neuropathic MPS II can live into their 60s, depending on disease severity [5,14]. Relying on clinical diagnosis can be difficult because infants with MPS II generally appear healthy at birth, and many signs and symptoms overlap with other childhood illnesses [15].

Early treatment initiation in MPS II is imperative because once symptoms manifest, most are not reversible, especially in the brain and skeletal system [16]. Delayed initiation of treatment is associated with more severe and life-limiting disease [16,17]. Studies comparing sibling outcomes support early initiation of treatment to improve outcomes [18]. Thus, diagnosis enabled by NBS and the timely and appropriate initiation of disease management will allow most affected newborns to achieve a better outcome than would be possible with a delayed clinical diagnosis [19]. This evidence led to the addition of MPS II to the US Federal Recommended Uniform Screening Panel (RUSP) in 2022 and its inclusion in many NBS programs in the US [18].

### 1.2. NBS Programs

In the US, NBS programs are state- or territory-based public health programs aimed at detecting treatable diseases soon after birth, enabling potentially timely and effective life-saving treatment [19]. The US Department of Health and Human Services (HHS) has previously recommended that US-based NBS programs screen for the diseases listed on the RUSP. Until recently, the RUSP was determined by the Secretary of HHS and administered by the Health Resources and Services Administration and the now-terminated Advisory Committee on Heritable Disorders in Newborns and Children. Inclusion of diseases is based on evidence submitted, interviews with experts, and discussion with committee members. Included within the decision matrix used by the committee are principles developed in 1968 through a commissioned report by the World Health Organization on the principles and practices of public health-based screening. These criteria, the so-called Wilson and Jungner Principles, include 10 characteristics of a disease that make it appropriate for population-based screening. These include aspects such as the disease being an important health problem with a recognizable latent or early symptomatic stage, the availability of diagnostic and treatment facilities, an effective treatment that can be administered presymptomatically, and economically balanced diagnostic and treatment costs [20]. Although RUSP decisions are not based on economic impact, analysis of cost to state programs and readiness for universal implementation are also elements of overall impact and implementation that are assessed.

The expansion of NBS programs to include more complex diseases has increased the need for improved infrastructure and coordination among governing bodies, health care providers (HCPs), and patients. Technological and therapeutic advances have led to the expansion of NBS programs, as more diseases now meet the Wilson and Jungner criteria. Indeed, there have been calls for modernization of NBS systems with an increased focus on infrastructure, system capacity, coordination of health care access and support, and the use of more molecular-based assays [21,22]. In addition, there is a growing focus on the ethical, legal, and social implications issues that are not fully covered by the Wilson and Jungner criteria. In particular, screening in the US currently operates under the legal doctrine, “*parens patriae,”* which means parental consent is not required for NBS to occur, as it has been determined that the importance of early detection and treatment of these diseases outweighs parental autonomy [23]. As a result, most NBS programs operate under quasi-mandates where the default is for NBS to occur unless parents choose to refuse screening on behalf of their newborn.

This model places increased responsibility on the state to provide adequate care following NBS and to educate medical providers and families about the diseases and their potential impact on the child and family [24]. These responsibilities are concurrent and require more robust infrastructure to improve communication of screen-positive results to parents and HCPs, to support affected individuals and families over the lifespan, and to incorporate trauma-informed care as families navigate a medical system poorly equipped for individuals with a rare disease [25]. There is also a need to improve data collection and sharing across NBS programs so that new knowledge gained through NBS can be more easily accessed and understood by HCPs and members of the public [26]. These data are needed for the long-term assessment of NBS programs to characterize the extent of improved health outcomes, identify barriers to care, and assess inequalities in NBS programs and the health care system.

All NBS programs should aim for universal access to NBS within their jurisdiction, as this has the potential to reduce inequalities in the health care system [27]. The successful implementation of a universal screening program requires a pre- and post-analytic infrastructure, beyond the analytic screening test, to achieve screening goals and provide support following screening [25]. Adequate infrastructure can ensure that confirmatory testing is conducted in a timely manner to reduce parental anxiety and that specialist care is available from the time of clinical referral [28,29]. With the growing number of diseases added to NBS panels, it is imperative that public health programs and health care systems have sufficient funding and staffing to meet the demands [26]. Reported barriers to implementing universal screening for MPS II in NBS programs include staffing issues, lack of funding, and the need for administrative approval [18].

However, universality does not in and of itself result in equity, and there are variations in the number of diseases screened and the methodologies used across states for NBS programs in the US, meaning that access to care for preventable disease is dependent on the geographical jurisdiction of an NBS program [26,30,31]. Some rare diseases can be more prevalent in certain ancestral groups [32], hence the lack of standardized testing across all states and territories has contributed to disparities among ancestral groups in the timeliness of diagnosis and treatment, testing optimization, and screening rates for individual diseases [27,31]. Despite these differences, no substantial ancestral or socioeconomic inequities in the overarching NBS policies across NBS jurisdictions have been identified [31].

### 1.3. Purpose of This Review

This narrative literature review by a multidisciplinary panel of NBS and MPS II experts aims to summarize the literature and provide practical recommendations on US-based NBS for MPS II and its diagnosis and clinical management. It is anticipated that these recommendations will help NBS program personnel, HCPs, and families work together to ensure the best possible outcomes for individuals with MPS II.

## 2. Methods

The sponsor invited authors to participate in the multidisciplinary expert panel based on their contributions to the field, ensuring a diversity of experience spanning clinical, patient advocacy, and research. Scientific literature was retrieved from the PubMed database and Google Scholar by searching for keywords including “MPS II,” “mucopolysaccharidosis type II,” “mucopolysaccharidoses,” “Hunter syndrome,” “newborn screening,” and “NBS.” Only articles written in English were included. Additional articles were found by searching reference lists and cited articles. Policy documents, resources from patient advocacy organizations, and other online sources were also included based on panel members’ professional knowledge and recommendations. Information on relevant clinical trials was retrieved from clinicaltrials.gov using search terms such as “MPS II,” “mucopolysaccharidosis II,” and “Hunter syndrome.” The panel selected articles for inclusion based on relevance, quality, and group discussion. The panel also provided practical examples and recommendations based on their professional experience.

## 3. MPS II NBS and Diagnosis

### 3.1. Practical Recommendations for NBS Programs

An enduring concern in NBS is the need to reduce false-positive screening results, which may cause distress within families and burden an already strained health care delivery system. Historically, post-analytic interpretation tools, such as the Collaborative Laboratory Integrated Reports in the US, have combined data from numerous laboratories to improve the predictive value of NBS tests and reduce false-positive rates [28]. However, a tiered approach to NBS, involving multiple biomarker assessments and testing strategies, is likely to be a more robust method to reduce the false-positive rate [28]. For example, a single-tier approach for MPS I resulted in unacceptably poor performance for programs owing to the detection of pseudodeficiency and other causes of decreased α-L-iduronidase activity unrelated to MPS I [33,34]. Implementing a second-tier approach for MPS I using GAG or molecular analysis has substantially reduced the false-positive rate compared with programs using only a single tier [33].

**Figure 2:**
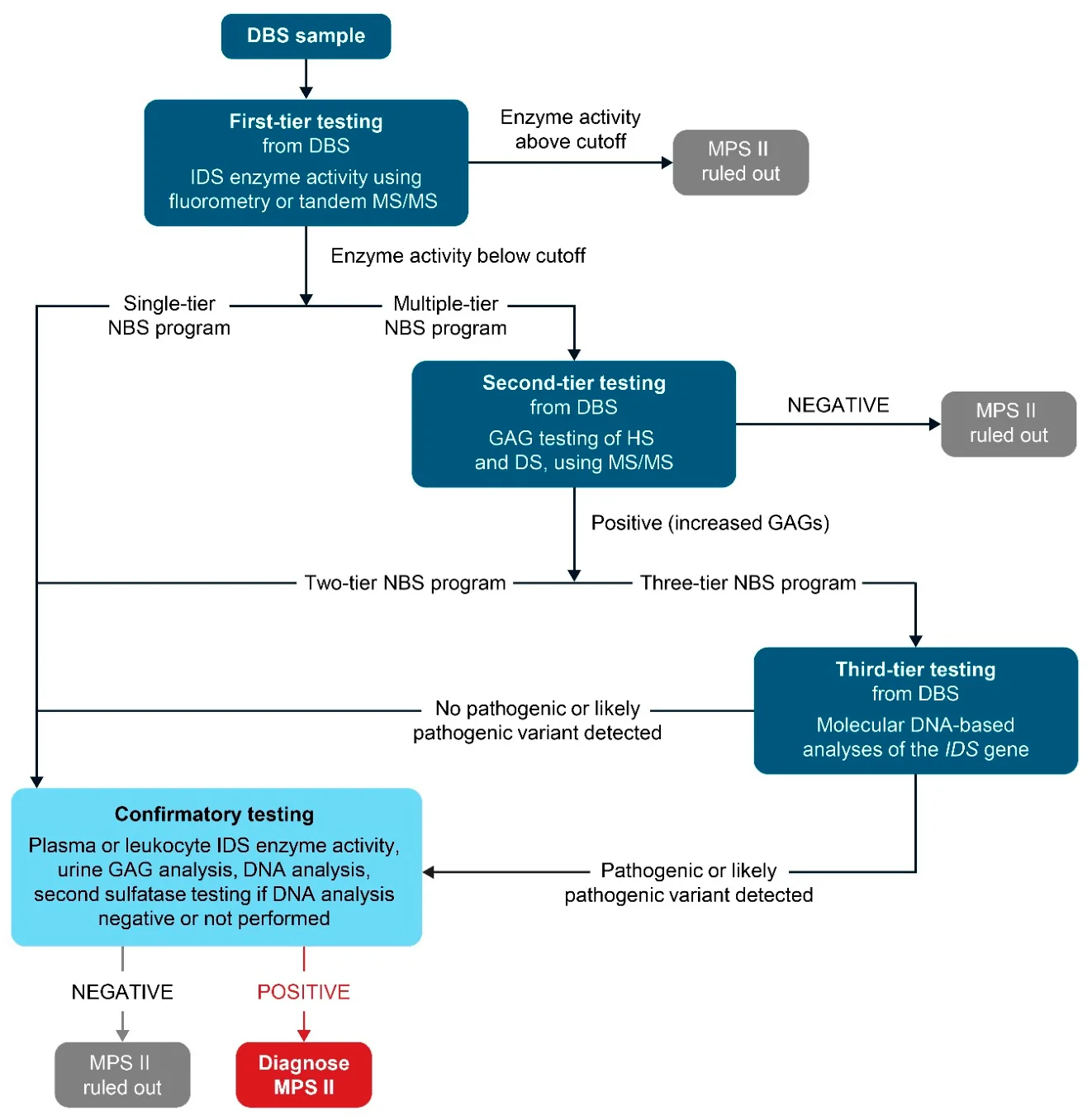
Testing strategies for identification of MPS II in NBS programs. Abbreviations: DBS, dried blood spot; GAG, glycosaminoglycans; MPS II, mucopolysaccharidosis type II; MS/MS, tandem mass spectrometry; NBS, newborn screening.

For MPS II NBS, initial biochemical testing uses a dried blood spot (DBS) to assess IDS activity by tandem mass spectrometry (MS/MS) or fluorometry (Figure 2 [1,35,36]). If the IDS activity is below the program’s set cutoff, second-tier testing should be undertaken to measure GAG levels from DBS using MS/MS [8]. These second-tier GAG targets should be either HS and DS (the internal disaccharide method) or the MPS II-specific endogenous non-reducing end oligosaccharide biomarker (the endogenous biomarker method) [35]. In a prior head-to-head comparison, both techniques were found to have comparable sensitivity, but the authors supported the endogenous biomarker method because it showed a greater differentiation between individuals with MPS II and healthy controls [35]. The authors noted that, for either technique, DBS GAG analysis requires mass spectrometers with higher sensitivity than is typically available in NBS laboratories. For this reason, NBS laboratories may consider outsourcing second-tier testing to reference laboratories. If DBS GAG levels are increased, the child should be referred for a clinical evaluation. This approach of primary IDS enzyme screening on DBS, followed by a second-tier analysis of GAGs, has been effective in identifying MPS II with an acceptable rate of false-positive results [35,37,38].

Follow-up confirmatory tests should include checking for a decrease in IDS activity from plasma or leukocytes and an increase in urine GAGs consistent with changes seen in MPS II [8]. The American College of Medical Genetics and Genomics (ACMG) ACT sheet and algorithm for MPS II also include arylsulfatase A leukocyte assays to help differentiate MPS II from Multiple Sulfatase Deficiency as part of a tiered approach to MPS II diagnosis [39,40]. Molecular analysis of the *IDS* gene may also be performed but can be complicated, as described below.

Some NBS programs also include DBS-based molecular DNA-based analyses, either as a primary screen, such as for Spinal Muscular Atrophy, or as a second- or third-tier test following an out-of-range biochemical test result [41]. Genetic analysis of the *IDS* gene may help confirm an MPS II diagnosis. However, there are >700 reported *IDS* gene variants associated with MPS II, with no highly recurring variants [42,43]. In a review of all *IDS* gene variants in MPS II published up to June 2023, 62.9% were classified as pathogenic and 35.4% as likely pathogenic, based on the ACMG/Association for Molecular Pathology criteria [43]. Most of these variants occurred in single families [43]. These features limit the robustness of genotype–phenotype correlations in MPS II and suggest that genetic testing alone may not be sufficient to establish an MPS II diagnosis. However, even in the absence of clear genotype–phenotype relationships, confirming the genotype can be beneficial in some instances, as this may predict a person’s response to therapy. For example, missense *IDS* genotypes have been associated with enhanced response to intrathecal idursulfase [44]. Additionally, approximately 15% of cases may be due to chromosomal-level changes, which are associated with a more severe disease course. Approximately half of these cases of chromosomal-level changes are a specific and recurrent rearrangement involving the *IDS* pseudogene, for which particular diagnostics are required, as this is not an allele that can be elucidated by Sanger sequencing.

Timeliness of these NBS processes is important to enable treatment initiation as soon as possible. The now-terminated Secretary’s Advisory Committee on Heritable Disorders in Newborns and Children provided timeliness goals for NBS, including communication of presumptive positive results to HCPs within the first 5 days of life for time-critical conditions (where acute symptoms or potentially irreversible damage can occur in the first week of life) and 7 days of life for other conditions, including MPS II [45]. Confirmatory testing should then be initiated as soon as possible. Once a diagnosis of MPS II is confirmed, treatment should be initiated as soon as possible for infants who are predicted to have a neuropathic presentation of MPS II and/or who have clinical signs/symptoms of the disease [46].

### 3.2. Practical Recommendations for HCPs

#### 3.2.1. Screening Algorithm

HCPs need to be aware of the benefits and limitations of the screening algorithm employed in their jurisdiction. First-tier enzyme activity assays based on fluorometry or MS/MS are fast, efficient, and inexpensive [47]. However, in some cases, pseudodeficiency may be detected in which the assayed enzyme activity is reduced but is not associated with a clinical phenotype [35]. The direct measurement of GAG levels in a DBS is a critical second-tier assay for MPS II and will substantially reduce the false-positive rate associated with assaying only DBS enzyme activity, because GAG evaluation will better distinguish MPS II disease samples from pseudodeficiency samples [48]. Additionally, pseudodeficiency of IDS is much more common than the true disease, highlighting the need for a two-tier testing approach for MPS II that relies on assessment of the stored metabolite [37].

#### 3.2.2. Communication of Screening Results

Positive screening results often come as a surprise to families, and families need to be made aware that most individuals with MPS II are asymptomatic at birth. Communication of these results should be given in a way that fosters understanding, empathy, and support. Results of a positive NBS test should be clearly communicated to parents in a timely manner and supported by both verbal and written information [28]. The recommended practice for informing parents includes using structured guidelines to inform communication, sharing the implications of the result, explaining the meaning of true- and false-positives, giving precise information on the next steps, and offering relevant printed materials or references to reliable websites [28]. Communication guides are available to assist HCPs in delivering this information [49,50,51]. Upon receiving positive NBS results, parents can experience a range of feelings, including nausea, shock, disbelief, fear, and sadness [52]. Some families have reported dissatisfaction with how results are initially communicated, and difficulties accessing reliable information at the right time [53,54]. However, because an unpleasant reaction is common and normal when receiving upsetting news, dissatisfaction from parents may not necessarily mean that the results were communicated inappropriately or inconsiderately. It is a nuanced challenge to find the right balance between emphasizing the urgency for further testing after a positive NBS result and avoiding unnecessary distress for the parents [28].

As an X-linked disease, it is important for families to understand that female relatives who do not display signs or symptoms of MPS II can still be carriers of the disease. This means that other family members could also be at risk, and an HCP or genetic counselor could assist in deciding whether other family members need to be informed and/or tested (see also “cascade testing” in Section 4.3: MPS II management—Practical Recommendations to Support Families).

### 3.3. Practical Recommendations to Support Families

Despite its importance, many families are unaware of or feel they do not know enough about NBS [55,56]. Therefore, it is important for NBS programs and HCPs to provide educational resources on NBS so that families understand its purpose and feel engaged and empowered when they choose to participate fully. Such educational materials may include printed informational leaflets and online resources, such as Baby’s First Test^®^ and the BabyCenter^®^ parenting platform [57]. Resources should be provided in a form that is accessible to families, recognizing that families may differ in their preferences for how they receive information.

Given the negative health impacts of delayed treatment for MPS II, it is important that all abnormal screening results are confirmed to be either true cases of MPS II or unaffected individuals with false-positive screening results. Between a positive screening result and a potential diagnosis, families should receive up-to-date information from an HCP knowledgeable about MPS II and informed about the recommended next steps in the diagnostic process.

## 4. MPS II Management

### 4.1. Practical Recommendations for NBS Programs

Although MPS II screening may be universal within a particular jurisdiction, this does not guarantee that all families can subsequently access appropriate care. It is therefore important for NBS programs and the surrounding health care systems to address potential disparities in health care access during program implementation. Sufficient NBS program infrastructure is needed to ensure that all newborns receive the benefits of NBS, including early detection and presymptomatic treatment. Disparities can arise across the NBS system and may include differences in access to health care and insurance, poor coordination of care, resource constraints, language barriers, transportation barriers, the cost of treatment, and perceptions about the disease. For those accessing Medicaid, there should be a plan or discussion of how these individuals will get the treatment and support they need. NBS programs should work with their Medicaid offices to discuss potential needs for out-of-network care, including across state or territory lines.

Longitudinal evaluation of the NBS system should take into consideration several factors. These include the diagnosed child’s access to health care services and a medical home practice model (a comprehensive, patient-centered, team-based model to provide health care); communication between HCPs, specialists, and the family; caregiver knowledge and skills, including self-advocacy; and access to up-to-date information for clinicians and families [58]. Additionally, to improve health care service delivery, data systems need to be kept up to date with details of health care utilization, treatments, disease outcomes, and quality of life. These metrics should take into account variations in practice patterns across geographic regions and socioeconomic status [58]. To help evaluate NBS programs, a long-term follow-up model, the Cares and Check Initiative, has been developed [25]. This initiative aims to identify general and disease-specific data points to facilitate care coordination, inform evidence-based treatment, enable quality improvement, and contribute to new knowledge discovery [25].

### 4.2. Practical Recommendations for HCPs

With the growth of NBS programs, there is a need to integrate effective public and professional education around NBS and rare diseases. The NBS Education Best Practices Framework has been designed to guide and monitor education programs across diverse cultures and audiences to improve health outcomes [59]. The satisfactory communication of positive NBS results can foster positive relationships, increase quality of care, enhance patient autonomy and adherence to health care guidance, and increase satisfaction [60]. Conversely, unsatisfactory communication can lead to relational issues and distrust, lower-quality care, over- or underuse of resources, dissatisfaction, and nonadherence, all of which result in short- and long-term negative impacts on children and families [61]. Clear, nontechnical communication about the disease, its prognosis, and available treatments should be delivered with empathy to help parents understand and cope with the situation [62]. Additionally, providing written, lay-accessible take-away materials can ensure that information is not forgotten [63].

When recommending approaches to initiating treatment for MPS II, HCPs should consider factors such as age, disease severity and prognosis, CNS involvement, and treatment benefits and risks [64]. If optimal treatment is constrained by cost or availability, HCPs should seek advocacy and social support mechanisms to help families access optimal care. Clinical practice recommendations advise that, due to the progressive nature of MPS II, treatment should be initiated as soon as possible after diagnosis, depending on the predicted phenotype [8,10]. As of July 2026, treatment options available in the US include enzyme replacement therapy (ERT), blood–brain barrier (BBB)-penetrant ERT, and hematopoietic stem cell transplantation (HSCT). Gene therapy, which is not commercially available at the time of writing, may become available in the near future. Each of these options has potential benefits and risks (Table 1).

For individuals with severe MPS II with symptoms or who are predicted to have severe MPS II based on their genotype, consensus practice recommendations suggest commencing intravenous (IV) ERT with idursulfase at the time of diagnosis [10]. Idursulfase is approved for MPS II in the US and other countries [65,66,67], with safety and efficacy (improved survival, physical function, and lung function) demonstrated in individuals with MPS II older than 5 years [65,68,69,95]. In addition, data from the Hunter Outcome Survey have demonstrated the efficacy and safety of idursulfase in younger children [70,96,97]. In a systematic literature review of 33 idursulfase studies, participants were similarly represented across age subgroups (infants, preschool children, school-age children, and adults) [72]. Twelve studies that included individuals aged <18 months demonstrated the potential benefits of idursulfase in this population, although the review authors noted that small sample sizes preclude definitive conclusions [72]. A number of case studies have also been published demonstrating the benefits of idursulfase when initiated in patients aged <18 months [74,75,98]. Indeed, long-term modeling of Hunter Outcome Survey data shows that the benefits of idursulfase after 8 years of treatment are greatest in children who start treatment at this age [96].

Idursulfase treatment does not result in sufficient enzyme crossing the BBB to significantly impact the CNS course of disease [17]. However, such patients will benefit from reduced somatic signs of MPS II, thereby experiencing positive therapeutic effects [17]. Alternatively, as shown in Table 1, one BBB-penetrant IV ERT, tividenofusp alfa, received accelerated approval in the US in March 2026. Other ERTs designed to cross the BBB are in clinical development in the US (pabinafusp alfa, which is approved in Japan) or under investigation (verenafusp alfa, which is approved in Russia). Additionally, intrathecal administration of idursulfase has been evaluated but failed to obtain approval from the US Food and Drug Administration; a phase 2/3 randomized trial of intrathecal idursulfase in individuals aged >3 years with MPS II and mild-to-moderate cognitive impairment failed to meet its primary endpoint of improved global intellectual ability at Week 52 [99]. However, idursulfase can also be delivered via the intracerebroventricular route, and idursulfase beta is approved for this route of administration in Japan [100].

All forms of ERT require frequent administration, which may be burdensome for patients over the long term [64,65]. A possible alternative to ERT for the neuropathic presentation of MPS II would be early presymptomatic HSCT. In HSCT, the recipient receives cells that produce the functional IDS enzyme [18]. An HSCT has the potential to be an effective one-time treatment [18,64,86,87]. However, it is associated with a high mortality risk in patients with MPS II (8% due to transplantation-associated complications [86]), and there is no clear consensus for HSCT in neuropathic MPS II [10,87] as there is for neuropathic MPS I. Another potential one-time treatment that is under investigation is RGX-121 gene therapy, which involves intracisternal or intracerebroventricular administration. There are also multiple clinical trials underway, which may be an option for some individuals (Supplementary Table S1, https://clinicaltrials.gov accessed on 2 July 2026).

Overall, it is important for HCPs to consider the availability of treatment options in their country. Standard IV ERT, such as idursulfase, is currently the only treatment with broad global availability, with approvals across major regions including North America, Europe, and Asia (Table 1). HSCT is also a treatment option; however, its use may be limited by the need for specialist centers and donor availability. Some newly emerging treatments have limited availability at the time of writing but may become more widely available in the future.

Given the potential approval and future availability of multiple treatment modalities, along with the advantages and disadvantages of each, it is important to consult a specialist in MPS II. Guidance is needed to help families make fully informed decisions about MPS II treatment options. It is essential for HCPs to maintain open lines of communication with parents and to outline the benefits and limitations of current and future treatment options, including those being evaluated in clinical trials.

### 4.3. Practical Recommendations to Support Families

Providing care for individuals with MPS II has significant impacts on caregivers, affecting their daily living and their social, emotional, psychological, and physical health [101]. Parents and caregivers often experience feelings of depression, anxiety, stress, and anger [63]. A diagnosis of a rare disease can also impact siblings, who may experience a complex range of emotions, including guilt, pride, worry, and sadness, while their life is affected by the care needs of their sibling [52,63]. The impacts of a diagnosis can range from bringing families closer together to straining familial relationships [52]. The parent–child relationship can also be affected, with some parents finding difficulty bonding with a child with a rare disease, and other parents find themselves being overprotective [52]. Circumstances often require parents and families to make huge sacrifices to care for a child or children with a rare disease, leading to social isolation and/or exclusion and financial difficulties [63]. Hence, it is essential for HCPs to develop a trusting and supportive relationship with families that allows them to discuss and express their feelings on such challenges.

Psychosocial support should be provided at the initial diagnosis to address psychological stress. This may include referrals to mental health professionals or to specialized support groups, especially those within the advocacy realm that have defined family support programs with experience in supporting families experiencing a new NBS-based diagnosis [62]. Providing comprehensive psychosocial support to families can improve coping mechanisms and emotional well-being, which enhances family resilience [62]. Online and in-person support groups, such as patient advocacy groups, can assist parents to meet with other families in similar situations, thus providing essential peer support [63]. Such timely support and intervention have been shown to limit the psychological impact that families experience when a child is diagnosed with a rare disease [63].

Finally, families should be offered the opportunity to speak with their HCP or a genetic counselor to help them decide whether other relatives need to be informed and/or tested for MPS II. This process is called cascade testing and is important because MPS II presentations are typically similar among family members, so it may help predict the severity and manifestations of MPS II [2,11,12,13]. Cascade testing may also help identify whether any other relatives are carriers of MPS II, which may assist with family planning.

## 5. Future of MPS II Screening, Diagnosis, and Disease Management

### 5.1. Considerations for NBS Programs

With the addition of MPS II to the RUSP in 2022, more states are anticipated to implement NBS for MPS II. Compared with clinical identification, NBS for MPS II is predicted to identify approximately twice as many MPS II cases [102]. This predicted increase in cases brings opportunities and challenges. Opportunities include the ability to better estimate MPS II prevalence, gather more information on disease progression, investigate genotype–phenotype correlations, and assess the impact of early treatment initiation. Challenges include the need for increased infrastructure and staffing to conduct screening tests, ensure equitable access to care, provide guidance for HCPs and families, provide psychosocial support, and conduct long-term follow-up and assessment of individuals with MPS II.

Other considerations for future NBS programs include technological advancements and increased adoption of techniques such as whole-genome sequencing. New technologies can bring potential benefits, as well as potential challenges. Benefits may include more cost-effective and accurate testing techniques. However, the utility of these would need to be assessed on an individual basis for the specific disease. For example, the utility of whole-genome sequencing in MPS II may be limited by the lack of robust genotype–phenotype correlations.

### 5.2. Considerations for HCPs

Now and into the future, HCPs will need to communicate effectively and empathetically with individuals with MPS II and their families. Families of individuals with rare diseases are becoming increasingly proactive in investigating and sharing information about their diseases, and one opportunity for HCPs to show their support is to acknowledge and express interest in the information families have found online about MPS II. In a survey of parents of children with rare diseases, some reported that their HCP did not seem interested in the information they had found online, suggesting this may be a missed opportunity to connect with families.

As outlined in previous sections, there is an array of potential treatments for MPS II, each with its own advantages and considerations. Consulting an MPS II specialist to support treatment recommendations will remain important to ensure that individuals with MPS II can access the most effective treatments as new treatments become available.

### 5.3. Considerations to Support Families

Helping families connect with support and patient advocacy groups will remain of key importance. Information on patient support groups was found to be one of the most frequently researched topics on the internet among parents of children with rare diseases, illustrating the need to connect with others in a similar situation.

With advances in technology, the way families find and consume medical information is evolving, with surveys showing a preference for online information and support, including parenting websites and social media. Both NBS programs and HCPs should deliver information in ways that reach individuals with MPS II and their families, including social media. Additionally, families of individuals with rare diseases are increasingly sharing information online with other families [103]. Individuals with rare diseases, advocates, and caregivers are also predicted to have an increasing impact on education, research, and legislation in the future [104]. Hence, NBS programs and HCPs may have a role in ensuring that families are aware of credible sources of information and that what they share is accurate.

## 6. Conclusions

This review presents information and practical recommendations on US-based MPS II screening, diagnosis, and management from a panel of MPS II experts. Recommendations for NBS programs include aiming for universal access to NBS within their jurisdictions, implementing tiered testing to improve screening accuracy, and providing infrastructure for confirmatory testing and post-screening support for families. Recommendations for HCPs include communicating test results empathetically alongside verbal and written information, allowing families to express their feelings, and consulting an MPS II specialist to support treatment recommendations. NBS programs and HCPs should work together to ensure positive screening results are communicated effectively and to provide equitable access to treatment and long-term care. The information in this review may also assist in educating and empowering families to make the best treatment/disease management decisions for their relative diagnosed with MPS II and to communicate effectively with their HCP.

In conclusion, through an adequately resourced and effectively coordinated effort by NBS programs and HCPs, current technologies and therapies have the potential to deliver the best possible outcomes for individuals with MPS II.

## Figures and Tables

**Table 1:** Benefits and considerations for MPS II treatment options.

Treatment	Description	Potential Benefits	Treatment Considerations	Regulatory Status as of July 2026
ERTs				
IV idursulfase	IV administration of recombinant IDS [65,66]	For those with symptoms or predicted severe MPS II, consensus practice recommendations suggest commencing idursulfase at the time of diagnosis [10] Safety and efficacy established for individuals aged over 5 years [65,66,67,68,69,70,71] Case studies and other reports of positive outcomes in children under 5 years of age [72,73], and infants under 18 months of age [74,75] Home treatment is available [10]	Black box warning for anaphylaxis [65] Although generally manageable and may become less frequent over time [72,76]; physicians should be familiar with the timing, nature, and management of IRRs Cognitive manifestations generally do not improve, as idursulfase cannot cross the BBB in significant amounts [10,72] Weekly IV administration over 3 to 4 h, which places a high access and logistic burden on patients [64]	Approved in the US, Europe, and other countries [65,66,67]
IV pabinafusp alfa (JR-141)	Human IDS fused to an anti-human transferrin receptor antibody designed to cross the BBB [77]	Phase 1/2 and 2/3 trials in Japan have shown the crossing of pabinafusp alfa into the CSF, resulting in a reduction in GAG accumulation and HS levels [77] Neurocognitive changes in patients with neuropathic MPS II appeared to be stabilized over the 52-week phase 2/3 trial of 28 patients of any age [77]	The 52-week study period in the phase 2/3 study was not considered sufficient to conclude there is a positive neurocognitive effect due to the progressive neurodegenerative nature of MPS II and long neurodevelopmental timeline in children [77] Weekly administration [78]	Approved in Japan [78]
Tividenofusp alfa (DNL310)	IV administration of a fusion protein consisting of IDS fused to a transport vehicle designed to cross the BBB via the transferrin receptor [79,80]	Potential to improve cognitive manifestations, as well somatic involvement [79,80]	Weekly administration [79]	Approved in the US * [81]
Verenafusp alfa (GNR-055)	IV administration of a recombinant modified ID2S designed to cross the BBB [82]	Potential to prevent neurodegeneration and cognitive deficit due to the ability to cross the BBB [82]	Weekly administration [82]	Approved in Russia [83]
Fetal ERT	Recombinant ERT infused through the umbilical vein of the fetus [84]	Potential to prevent organ and tissue damage before birth	Prenatal diagnosis required, which may not be possible for families without a known history of disease [84,85]	Investigational
Procedures				
Allogenic HSCT	The recipient receives cells producing functional IDS enzyme from a donor [18]	Potential to improve CNS symptoms, organ function, and daily living activities [18,64,86,87] One-time therapy [64] May be more cost-effective than ERT [88]	Data on HSCT for MPS II are limited and variable [87], and there is no clear consensus on when HSCT should be used [10,87] Not suitable for all patients [18,64] High mortality risk (8% mortality reported among patients with MPS II) [86] Chance of graft failure [87] Limited by donor availability [64]	Available
HSCT cell gene therapy	Autologous CD34+ cells are transduced with an ApoEII ligand tagged human *IDS* gene [87,89]	The ApoEII ligand tag may assist with efficient BBB transcytosis immediately on stem cell engraftment and subsequent engrafted microglial repopulation of the CNS [89] Use of autologous cells typically reduce mortality risk, compared with allogenic HSCT [87]	Similar risks to allogenic HSCT, including requirement for chemotherapeutic conditioning for successful engraftment [87]	Investigational
Gene therapy				
Clemidsogene lanparvovec (RGX-121)	*IDS* gene delivered to cells in the CNS [90]	Potential one-time treatment [90] Potential to improve cognitive manifestations through intracisternal and intracerebroventricular administration [90,91,92]	Requires immune suppression [93] Some individuals require weekly ERT after gene therapy procedures [90]	Investigational [94]

* Granted under accelerated approval on 25 March 2026, based on Phase 1/2 trial results [81]. Abbreviations: BBB, blood–brain barrier; CNS, central nervous system; CSF, cerebrospinal fluid; ERT, enzyme replacement therapy; GAG, glycosaminoglycans; HS, heparan sulfate; HSCT, hematopoietic stem cell transplantation; IDS, iduronate-2-sulfatase; IRR, infusion-related reaction; IV, intravenous; MPS II, mucopolysaccharidosis type II.

## Data Availability

No new data were created or analyzed in this study. Data sharing is not applicable to this article.

## Supplementary Materials

The following supporting information can be downloaded at https://www.mdpi.com/article/10.3390/ijns12030066/s1, Supplementary Table S1. Clinical trials for the treatment of MPS II.
