# Supplementary material for: Mucopolysaccharidosis Type II Screening, Diagnosis, and Management: A Literature Review and Practical Recommendations for Newborn Screening Programs and Health Care Providers to Support Families and Improve Outcomes
Source: Int J Neonatal Screen. 2026 Aug 12;12(3):66. doi: 10.3390/ijns12030066 (PMC13510929; doi:10.3390/ijns12030066)
Supplement: Supplementary file 1 [file IJNS-12-00066-s001.zip › IJNS-4287600-supplementary.pdf]

Supplementary Table S1. Clinical trials for the treatment of MPS II.

Product and company / sponsor	NCT# and study name	Ph	MPS II type	Age	Endpoints	N	Dates and status as of July 2026
				CNS penetrant IV ERT			
Tividenofusp alfa (DNL310) from Denali Therapeutics Inc.	NCT04251026 DNLI-E-0002	1/2	Non-neuropathic and neuropathic	<18 years	1° TEAEs, GAG concentration in urine, IRRs, changes in concomitant medications<br>2° HS concentration in CSF and urine, VABS subscales, VABS ABC score, PK, ADAs, liver volume	47	July 16, 2020 to February 2031<br>Active, not recruiting
	NCT06075537 (extension of Study DNLI-E-0002 and DNLI-E-0007)	2/3	Non-neuropathic and neuropathic	<18 years	1° TEAEs, GAG concentration in urine, IRRs<br>2° HS concentration in CSF and urine, DS in urine, VABS-III, BSID-III, 6MWT, liver and spleen volume, CaGI-C	99	September 20, 2023 to June 2027<br>Enrolling by invitation
	NCT05371613 COMPASS, DNLI-E-0007	2/3	Non-neuropathic and neuropathic	≥2 to <26 years	1° HS concentration in CSF, VABS-III<br>2° BSID-III, VABS-III ABC score, serum neurofilament light chain, 6MWT, HS and DS concentration in urine, liver and spleen volume, CaGI-C	63	July 21, 2022 to December 2027<br>Recruiting

Pabinafusp alfa (JR-141) from JCR Pharmaceuticals	NCT04348136 (extension of study JR-141-301)	2/3	NS	NS	1° AEs, vital signs, Kyoto Scale of Psychological Development, VABS-II, Bayley-III or KABC-II, HS/DS levels (serum, urine, CSF), liver and spleen volume, cardiac function, 6MWT, joint range of motion	27	September 1, 2019 to March 31, 2030 Active, not recruiting
	NCT03359213 JR-141-BR21	2	NS	≥0 years	1° AEs<br>2° PK, urinary and serum HS and DS, liver and spleen volumes, and left ventricular mass	18	July 26, 2018 to October 4, 2019 Completed
	NCT03708965 (extension of JR-141-BR21)	2	NS	0 and older	1° AEs<br>2° HS and DS in urine and serum, liver and spleen volumes, echocardiography	19	January 1, 2019 to December 31, 2027 Active, not recruiting
	NCT03568175 JR-141-301	2/3	NS	NA	1° HS Levels in CSF<br>2° HS/DS levels (serum, urine, CSF), liver and spleen volume, cardiac function, 6MWT, joint range of motion, Kyoto Scale of Psychological Development 2001, VABS-II	28	August 1, 2018 to February 20, 2020 Completed
	NCT04573023 (STARLIGHT)	3	NS	Child, adult, older adult	1° BSID-III, HS levels in CSF<br>2° BSID-III, VABS-II, change in liver and spleen volume	86	February 14, 2022 to October 31, 2027 Active, not recruiting

	NCT05594992 Extension of JR-141-GS31	3	NS	Child, adult, older adult	1° HS levels in CSF, neurocognitive changes by BSID-III, KABC-II, VABS-II, and Wechsler<br>2° liver and spleen volume, 6MWT	80	February 22, 2023 to February 28, 2028<br>Enrolling by invitation
Gene/cell therapy							
Clemidsogene lanparvovec (RGX-121) from REGENXBIO, Inc.	NCT07236606	3	Neuropathic	≥4 months to <5 years	1° neurodevelopmental function by BSID-III and KABC-II scales, TEAEs and SAEs<br>2° GAG concentration in CSF, urine and plasma, I2S concentration in CSF and plasma, VABS-II score, change in brain volume, gray and white matter, auditory capacity by ABR, safety TEAEs, laboratory evaluations, vital signs, physical examinations and neurological assessments, change from baseline in BSID-III and KABC-II scores	2	November 25, 2025 to May 30, 2031<br>Active, not recruiting
	NCT03566043 CAMPSIITE, RGX-121-101	1/2/3	Neuropathic	≥4 months to <5 years	1° TEAEs, CSF GAG levels, BSID-III or MSEL<br>2° TEAEs, GAG levels and IDS activity, BSID-III, KABC-II, MSEL,VABS-II, brain size	48	September 27, 2018 to August 2025<br>Active, not recruiting

	NCT04571970<br>RGX-121-1102	1/2	Neuropathic	≥5 to <18 years	1° AEs<br>2° TRAEs, GAG levels, IDS activity, BSID-III, MSEL, VABS-II	6	March 11, 2021 to May 23, 2024<br>Completed
Hematopoietic stem cell gene therapy using CD34+ cells with ApoEII tagged IDS gene from University of Manchester	NCT05665166<br>R125432	1/2	Neuropathic	≥3 and ≤22 months	1° AEs, presence of replication competent virus and integration events in the leukocytes<br>2° HS Levels in CSF, plasma and urine, GAG levels in urine, IDS enzyme activity in plasma, total leucocytes, CSF and bone marrow, VCN in total leukocyte and bone marrow, proportion of cells containing *IDS.ApoEII* gene in bone marrow colony forming units, BSID-III or KABC-II, VABS-III<br>Other: DS in CSF, plasma and urine, IDS enzyme activity in CD3+, CD15+ and CD19+ cells, apnea-hypopnea index, anti-IDS antibodies in blood and CSF, hearing, height, exploratory biomarkers, BSID-III or KABC-II, VABS-III, parental reported observations	5	June 1, 2023 to July 2028<br>Active, not recruiting

DUOC-01 cell therapy (UCB-derived oligodendrocyte-like cells) as an adjunct to UCBT for IMD including MPS II, Joanne Kurtzberg, MD, Duke University	NCT02254863	1	NS	1 week to 22 years	1° Neurological and infusional toxicity<br>2° Standard of care follow up evaluations	40	September, 2014 to October, 2026<br>Recruiting
Reduced intensity conditioning for HSCT for IMD including MPS II, Paul Szabolcs, University of Pittsburgh	NCT01962415	2	NS	2 months to 55 years	1° Post-HSCT mortality, neurodevelopmental milestones, immune reconstitution, incidence of infections and GVHD<br>2° donor cell engraftment, enzyme level, neutrophil and platelet recovery, incidence of grade 3-4 organ toxicity and late graft failure	100	February 4, 2014 to November, 2027<br>Recruiting

Busulfan- and fludarabine-based conditioning regimens with busulfan TDM for HSCT for IMD including MPS II, Masonic Cancer Center, University of Minnesota	NCT02171104	2	Predicted to have neuropathic	0 to 55 years	1° proportion of participants with high-level donor cell engraftment<br>2° Incidence and severity of GVHD, incidence of TRM, regimen-related toxicity and post-HSCT disease changes	100	July 10, 2014 to July 14, 2029<br>Active, not recruiting
Anti-inflammatory							
Adalimumab from Lundquist Institute for Biomedical Innovation at Harbor-UCLA Medical Center	NCT03153319<br>31041-01	1/2	NS	≥5 years	1° Pain, adalimumab trough<br>2° Joint range of motion, pain, TEAEs	14	June 5, 2017 to February, 2027<br>Active, not recruiting
ERT							

Elaprase from Takeda	NCT05494593	4	NS	<6 years	1° rate of development of anti-idursulfase antibodies<br>2° correlation between ADA response, IDS gene mutations and clinical outcomes, change from baseline in urine GAG levels normalized to urine creatinine, urine GAG levels normalized to ULN, and liver volume	5	February 28, 2023 to August 29, 2025<br>Withdrawn due to enrollment challenges
	NCT05058391<br>TAK-665-4001	4	NS	Any age	1° AEs, TEAEs, IRRs<br>2° Forced vital capacity, 6MWT, cardiac left ventricular mass index, ejection fraction, liver and spleen volume, GAG levels in urine, global joint range of motion, height, weight, HRQoL, CHAQ	5	April 21, 2022 to April 18, 2024<br>Completed
Elaprase with Idursulfase-IT (HGT-2310) from Takeda (Shire)	NCT02412787 (extension of NCT02055118)<br>SHP609-302	2/3	NS	NS	1° AEs, vital signs, enzyme concentration in serum and CSF, GAG concentration in CSF and urine, anti-idursulfase antibodies in CSF and serum<br>2° DAS-II, BSID-III, VABS-II, brain structure volume	56	April 14, 2015 to April 18, 2024<br>Completed
	NCT01506141<br>Extension of (NCT00920647)<br>HGT-HIT-046	1/2	NS	3 to <18 years	1° TEAEs<br>2° PK, GAG levels in CSF and urine	15	August 1, 2010 to April 30, 2024<br>Completed

	NCT06031259 (extension of NCT02412787 and NCT01506141)	2/3	NS	3 to 19 years	1° TEAEs, AEs related to treatment, device surgical procedures, IT administration processes, IDDD and IV infusion	6	March 5, 2024 to January 1, 2029 Active, not recruiting
Elaprase with Idursulfase-ICV (GC1123, ICV-Hunterase) from GC Biopharma Corp	NCT05422482	1	Neuropathic	18 months to 18 years	1° TEAEs, SAEs, presence of abnormal echocardiography results<br>2° PK/PD characteristics of GC1123, presence of ADAs and neutralizing antibodies	12	September 20, 2022 to June 2027 Active, not recruiting
GC1111 from Green Cross Corporation	NCT03920540 GC1111_P3	3	NS	≥5 years	1° 6MWT	32	March 9, 2017 to February 15, 2022 Completed
	NCT02663024 GC1111B_P2	2	NS	≥5 to ≤35 years	1° GAG concentration in urine<br>2° 6MWT, liver and spleen, volume, AEs, safety evaluations, immunogenicity, PK	20	December 2016 to June 2020 Unknown status
Verenafusp alfa (GNR-055) from AO Generium	NCT04539340 IDB-MPS-I	1	Healthy volunteers	≥18 to ≤50 years	1° AEs<br>2° PK	20	February 28, 2020 to September 8, 2020 Completed

	NCT06475404<br>IDB-MPS-I02	1	Healthy volunteers	≥18 to ≤45 years	1° AEs<br>2° PK	18	April 6, 2023 to September 20, 2023<br>Completed
	NCT05208281<br>IDB-MPS-II-III	2/3	NS	NS	1° AEs, GAG concentration in urine<br>2° GNR-055 concentration in serum, GAG concentration in CSF and serum, large joint range of motion, liver and spleen volume, 6MWT, left ventricular mass, lung forced vital capacity, neurocognitive function, brain structure, serum and CSF neuromarkers	32	November 30, 2021 to March 2028<br>Recruiting
Fetal ERT, University of California, San Francisco	NCT04532047<br>PEARL	1	NS	8 0/7 to 34 6/7 weeks gestation	1° AEs, dose, GAG in urine, improvement/resolution of hydrops<br>2° antibody levels<br>Other: number of participants that show functional cardiac, growth, mobility, and neurocognitive function	10	July 1, 2021 to July 31, 2032<br>Recruiting

All data are from: https://clinicaltrials.gov, accessed August 21, 2025 and updated December 2, 2025, and July 2, 2026.

Abbreviations: 6MWT, 6-minute walk test; ABC, adaptive behavior composite; ADA, anti-drug antibody; AE, adverse event; BSID, Bayley Scales of Infant and Toddler Development; CaGI-C, Caregiver Global Impression of Change; CD, cluster of differentiation; CHAQ, Childhood Health Assessment Questionnaire; CNS, central nervous system; CSF, cerebrospinal fluid; DAS, disease activity score; DS, dermatan sulfate;

ERT, enzyme replacement therapy; GAG, glycosaminoglycans; GVHD, graft-versus-host disease; HRQoL, health-related quality of life; HS, heparan sulfate; ICV, intracerebroventricular; IDDD, intrathecal drug delivery device; IDS, iduronate-2-sulfatase; IMD, inherited metabolic diseases; IRR, infusion-related reaction; IT, intrathecal; IV, intravenous; KABC, Kaufman Assessment Battery for Children; MPS II, mucopolysaccharidosis type II; MSEL, Mullen Scales of Early Learning; NS, not stated; Ph, Phase; PK, pharmacokinetics; TDM, therapeutic drug monitoring; TEAE, treatment-emergent adverse event; TRAE, treatment-related adverse event; TRM, transplant-related mortality; UCB, umbilical cord blood; UCBT, umbilical cord blood transplantation; ULN, upper limit of normal; VABS, Vineland Adaptive Behavior Scale; VCN, vector copy number.
